# Retraction: Severe Acute Respiratory Syndrome Coronavirus 2 (SARS-CoV-2) Infection Mimicking as Pulmonary Tuberculosis in an Inmate

**DOI:** 10.7759/cureus.r34

**Published:** 2021-08-31

**Authors:** Hina Akbar, Rehan Kahloon, Sobia Akbar, Arslan Kahloon

**Affiliations:** 1 Internal Medicine, University of Tennessee Health Science Center, Memphis, USA; 2 Internal Medicine, Regional One Hospital, Memphis, USA; 3 Cardiology, Erlanger Health System/University of Tennessee College of Medicine, Chattanooga, USA; 4 Internal Medicine, Postgraduate Medical Institute, Lahore, PAK; 5 Gastroenterology, Erlanger Health System/University of Tennessee College of Medicine, Chattanooga, USA

This article has been retracted at the request of the authors. Their full statement is included below:

Our case report, "Severe Acute Respiratory Syndrome Coronavirus 2 (SARS-CoV-2) Infection Mimicking as Pulmonary Tuberculosis in an Inmate" contains some inadvertent errors and lacks some pertinent patient information that could have been added to the manuscript. Information about COVID-19 was rapidly evolving at the time the article was published so we are unable to confirm if the patient's presenting symptom of hemoptysis was from COVID-19. We believe the report may not accurately represent the patient’s clinical course, diagnosis and treatment and there is a chance that the reader can get confused and possibly misinterpret our case. We think that a more thorough review of literature and a more detailed discussion with other care team members/consultants would have greatly improved the quality of our article.

We are unsatisfied with the quality of our work and we think that the report in its current form is not up to the mark or as scientifically sound as we intended our work to be. As COVID-19 is a topic of great interest with a large reader population, we want readers to receive our work in the highest possible standard. We hope to publish a revised article which is higher quality, error-free and without any ambiguity. For these reasons, we would like to request a retraction of the article in order to replace it with an article that meets the best standards of our own work, the journal and the scientific community as a whole.

